# Barriers and facilitators to cognitive impairment screening among older adults with diabetes mellitus and hypertension by primary healthcare providers in rural Uganda

**DOI:** 10.3389/frhs.2023.1172943

**Published:** 2023-05-30

**Authors:** Moses Muwanguzi, Celestino Obua, Samuel Maling, Wilson Wong, Judith Owokuhaisa, Edith K. Wakida

**Affiliations:** ^1^ Faculty of Medicine, Mbarara University of Science and Technology, Mbarara, Uganda; ^2^ Department of Pharmacology and Therapeutics, Mbarara University of Science and Technology, Mbarara, Uganda; ^3^ Department of Psychiatry, Faculty of Medicine, Mbarara University of Science and Technology, Mbarara, Uganda; ^4^ Department of Medical Education, California University of Science and Medicine, Colton, CA, United States; ^5^ Department of Community Health, Mbarara University of Science and Technology, Mbarara, Uganda

**Keywords:** cognitive, screening, primary health care, older adults, hypertension, diabetes mellitus, dementia

## Abstract

**Background:**

The burden of non-communicable diseases and cognitive impairment exhibit a linear rise in sub-Saharan Africa due to the increase in life expectancy. Non-communicable diseases like diabetes mellitus and hypertension increase the risk for cognitive impairment. To improve our understanding of the underpinnings of the cognitive impairment screening, this study explored the barriers and facilitators of routine cognitive impairment screening in a primary healthcare setting guided by the Capacity, Opportunity, Motivation Behavioral change (COM-B) model.

**Methods:**

This was a descriptive qualitative study with primary healthcare providers who provide care to older adults with diabetes mellitus and hypertension at three primary healthcare centers in Mbarara district southwestern Uganda. In-depth interviews were conducted using a semi structured interview guide. Interviews were audio-recorded, transcribed verbatim, and analyzed using the framework approach along the COM-B components. Each COM-B component factors were categorized as barriers and facilitators.

**Results:**

We conducted 20 in-depth interviews with clinical officers, enrolled nurses, and a psychiatric nurse. The questions were guided by the Capacity, Opportunity and Motivation Behavioral change (COM-B) framework to identify barriers and facilitators to cognitive impairment screening. The factors that negatively affected the screening were considered as barriers, while the positive as facilitators. Capacity related barriers to cognitive impairment screening included chronic understaffing, primary healthcare provider non-involvement, lack of training/skills, lack of knowledge and awareness in screening, absence of caretakers, lack of patient awareness of cognitive problems; while facilitators were staff recruitment, primary healthcare provider involvement, and specialized training. Opportunity related barriers to screening included patient overload, infrastructure shortage, and time constraints. Motivation related barriers included lack of screening guidance and policy, while the facilitators were availability of mentorship programs for primary healthcare providers.

**Conclusions:**

Integrating cognitive impairment screening in primary health care requires engagement of relevant stakeholders with the focus on addressing implementation challenges through capacity development. Timely cognitive impairment screening at the first point of care initiates a cascade of interventions for timely enrollment into care, thus arresting the progress of cognitive impairment that leads to dementia.

## 1. Background

Cognitive impairment increases the risk of developing dementia and increases morbidity and mortality in the elderly (1). The World Health Organization (WHO) recognizes dementia as a public mental health priority and calls for early diagnosis, appropriate treatment and care (2). Due to the increasing burden of non-communicable diseases worldwide especially Diabetes Mellitus (DM) and Hypertension (HTN), and the increase in life expectancy, the prevalence of cognitive impairment has been projected to dramatically rise especially in low and middle income countries where early cognitive impairment screening is still underemphasized (3). Worldwide, health promotion has focused on early screening and timely initiation of appropriate management approaches (2).

Although early detection of cognitive impairment may not halt the onset of degenerative dementia, and the existing treatments cannot reverse its course, the health, psychological, and social benefits of early detection are important to make a screening program worthwhile (4). Effective uptake of guidelines and recommendations of clinical practice requires not only attitude and behavioral change by primary healthcare providers, but also structural modifications of the healthcare system and work environment through identifying and effectively addressing existing barriers (5).

Early screening and detection of mild cognitive impairment facilitates early involvement of the family and community in care along with promoting awareness on dementia as a pathological cognitive impairment rather than being considered as a normal aging process (6). In addition, early detection is important in developing relevant cognitive function rehabilitation plan and formation of a more patient-specific and caregiver support and follow-up plans (7). Post-diagnostic rehabilitation is a human right to all older people diagnosed with dementia, and has been shown to improve their quality of life and possibly slowing down dementia progression (8). This is essential in conservation of cognitive reserve hence retardation of the progression to severe forms of cognitive impairment.

Using the Capability, Opportunity, and Motivation to behavioral change framework (COM-B), we explored the barriers to, and the facilitators of screening for cognitive impairment among older adults with DM and/or HTN by primary healthcare providers in rural southwestern Uganda. Identifying and addressing the barriers to routine screening of cognitive impairment among older adults with DM and/or HTN along with leveraging the existing facilitators may go a long way in influencing primary healthcare providers’ adoption of the desired behavior.

### 1.2. Theoretical framework

**Figure 1:**
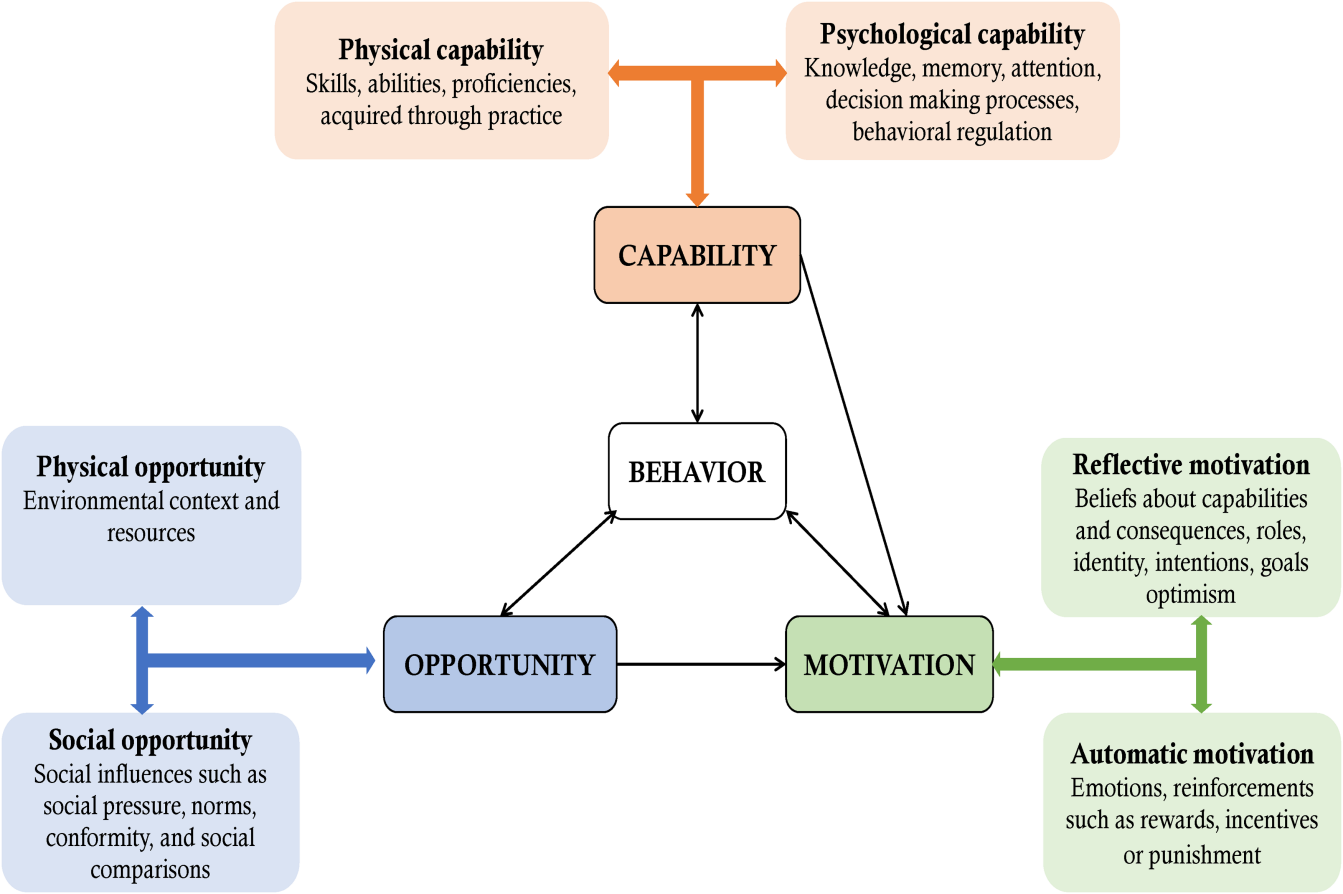
The capability opportunity motivation behavioral change model (9).

We used the theoretical model of behavior change which utilizes Capability, Opportunity and Motivation to understand behavior change (9). The COM-B theory postulates that for behavior change to take place, there should be interaction between one or more of the capability (physical and psychological) to carry out a behavior, opportunity (physical and social) and motivation (reflective and automatic) to perform a desired behavior (9) (Figure 1).

We characterized Capability as physical (skills, abilities, proficiencies) and psychological (knowledge, memory, behavioral regulation) abilities needed by the primary healthcare providers to screen for cognitive impairment; Opportunity as social (peer pressure, norms, conformity, and comparisons) and physical (environmental context and resources) influences to screening for cognitive impairment; and Motivation as reflective (beliefs about capabilities, roles, intentions), and automatic (emotions, reinforcements such as incentives or punishment).

## 2. Methods

### 2.1. Study design and setting

We conducted a qualitative exploratory study that used one-on-one semi-structured interviews to describe individual perspectives of the primary healthcare providers while in their routine clinical care setting. Our aim was to identify factors that influenced cognitive impairment screening among older people with DM and/or HTN by primary healthcare providers in primary healthcare facilities in southwestern Uganda. The qualitative interviews were guided by the COM-B framework. We approached this study from an exploratory perspective using a participant-oriented perspective for improved engagement, acceptability, and clinical outcomes. The study was designed by JO and MM in consultation with SM, EKW and CO.

The study was conducted at three primary healthcare facilities in Mbarara district which is approximately 270 kilometers (170 miles), by road, southwest of the capital city, Kampala (10). We purposively selected the primary healthcare facilities that offered diabetes and hypertension services. These facilities provided screening and diagnostic services as well as management and follow-up of patients with DM and/or HTN.

### 2.2. Study participants

The target study participants were clinical officers (diploma level training in clinical medicine), and nurses (general practitioners and psychiatric) directly involved in providing care to people with DM and/or HTN at the outpatients’ departments of participating primary healthcare facilities. All primary healthcare providers regardless of the years of experience at primary health care level or with screening for cognitive impairment were included. Study participants were recruited by the lead researcher (MM) through phone calls to schedule in-person interviews. The study purpose was introduced to the target participants and only those who provided written informed consent were included in the study.

### 2.3. Data collection tool

A semi-structured interview guide (Additional file 1) was developed corresponding to key COM-B domains as the *a priori* themes, with questions adapted to practice of cognitive impairment screening. The study tool was pretested with two nurses and one clinical officer at a primary healthcare facility not included in the main study. The purpose of the pretest was to ensure clarity of the questions; we used feedback from the interviews to refine the questions.

### 2.4. Data collection procedure

Data were collected was in March 2022. In-depth interviews were conducted by JO (background in nursing and skills in qualitative methods) and a research assistant (CA) with a public health background and experience in conducting qualitative research interviews. To address reflexivity, the JO (the corresponding author) conducted the initial interviews together with CA to ensure consistency in the conduct of the interviews. The rest of the interviews were conducted individually by JO and CA. There was no prior relationship between the participants and the interviewers (11). The participants were informed about the purpose of the study, namely to gain their perceptions about factors influencing their practice in cognitive impairment screening among older people with DM and/or HTN. All participants were assured about confidentiality of their responses and that any publications would be de-identified with respect to quotations from the interviews (12). Informed consent was obtained, and interviews were conducted in private spaces. Verbal consent was obtained to audio record the interviews, supported with field notes. A conversational approach was used with the participants during the interview, probing and motivating them to provide complete and accurate information (13) All interviews were conducted in-person, in English language (the official language in Uganda), and lasted for approximately 30 to 40 min.

### 2.5. Data management and analysis

All audio recorded interviews were transcribed verbatim by CA, and the transcripts reviewed by MM for accuracy (14), inserting notations for pauses, clarification of information and punctuations. All the transcripts were read and re-read by MM and JO to familiarize with the data and the overall meaning (15). Data were manually organized using a framework matrix (16) guided by the COM-B domains (capability, opportunity and motivation) as the broad themes. The rows were used for sub-themes (physical and psychological capability, social and physical opportunity; reflective and automatic motivation), while the columns represented the data sources (from probes, field notes), and responses from the participants (15). The content in the cells of the raw matrix was mapped to the COM-B domains to check that the responses were correctly placed under each category (16). Coding was done by MM and JO independently, and reviewed by EKW and CO for consistency checks to ensure rigor (17, 18). Where there was disagreement on content mapping, there was discussion and responses shifted to where they were most appropriate by consensus. MM and JO conducted the initial analysis of the data, shared and discussed the emerging themes with the rest of the authors.

## 3. Results

### 3.1. Characteristics of study participants

A total of 20 in-depth interviews were conducted, participants characteristics were stratified by age, gender, cadre and work experience. Of the 20 in-depth interviews conducted, 12 were with enrolled nurses (each with a diploma in nursing or certificate in comprehensive nursing), 7 with clinical officers (each with a diploma in general clinical medicine) and 1 with a psychiatric nurse (with a diploma in psychiatric nursing). The participants were aged between 24 years and 57 years. The majority of the primary healthcare providers were females (15/20), mostly nurses (13/20). The work experience ranged from 1 year to 18 years.

### 3.2. Barriers and facilitators to cognitive impairment screening

**Figure 2:**
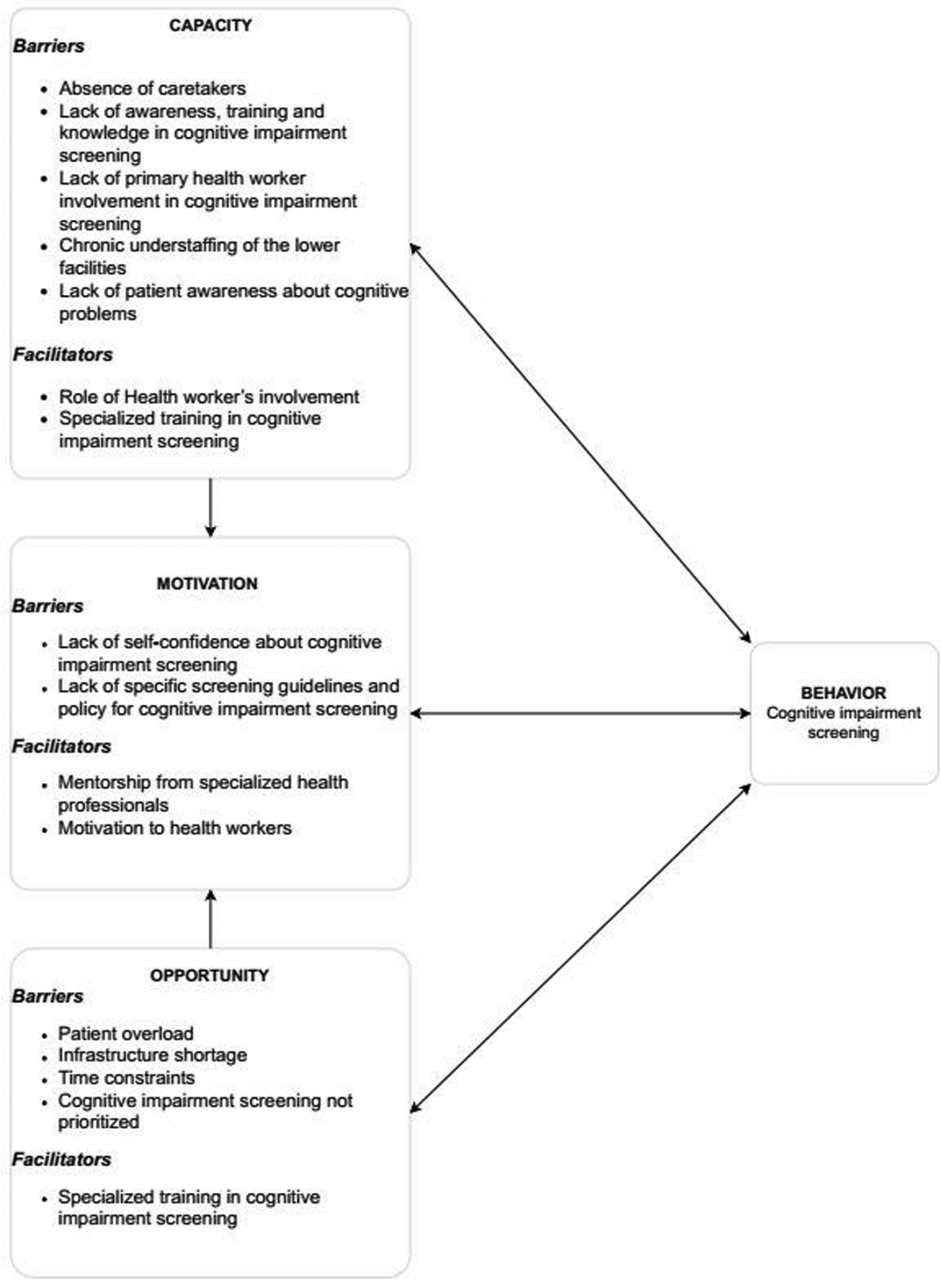
Barriers and facilitators to cognitive impairment screening of older adults with diabetes mellitus and hypertension mapped onto capability opportunity motivation behavioral change model.

The results were organized corresponding to three COM-B domains that guided the interview including (i) Capability—physical and psychological; (ii) Opportunity—physical and social; and (iii) Motivation—reflective and automatic. The factors that negatively affected the components of the COM-B framework were categorized as barriers and those that positively affected the components were grouped as facilitators (Figure 2).

## 4. Domain 1: capability

### 4.1. Physical capability

#### 4.1.1. Barriers

##### 4.1.1.1. Absence of care takers

Primary healthcare providers reported that majority of older adults came alone to seek health care, and that some of them did not provide adequate information about their cognitive functioning. Additionally, participants mentioned history taking as a common tool in screening and diagnosis for most illnesses. In this case, collateral history from care takers was mentioned to be relevant compared to the information given by the older adult alone. Absence of care takers therefore was reported to compromise the screening process of cognitive impairment.

“*Some patients do not have care takers, he or she is in his sixties, comes alone, she has come to get treatment, you may not get what you want exactly … is alone, sometimes you want someone to help you in taking history, to dig more, especially about issues happening at home. But, the muzeyi (old man) has come alone” **P2, 32 years old, Female.***

##### 4.1.1.2. Lack of awareness, training and knowledge in cognitive impairment screening

Participants reported lack of awareness, training and knowledge as barriers to screening for cognitive impairment. Some of them confessed that they had never heard about screening for cognitive impairment, while others believed that memory problems were part of the normal aging process. They reported no reason to give it attention and concentrated on other conditions that they believed posed a greater health threat.

“*For me, I have not received any training in cognitive impairment … so, how am I expected to know much about it? I may be knowing some things, but what I know may not allow me to do the screening well, as it should be … when I get an aging person with a memory problem … that's part of life caused by aging … yet we have serious illness here like malaria, HIV which are more serious and cause death. So, for me, I think that (cognitive impairment) doesn't cause serious health problems … many old people have memory problems but are not bed ridden” **P12, 40 years old, Female.***

##### 4.1.1.3. Lack of primary healthcare provider involvement in cognitive impairment screening

Most of the participants, irrespective of their years of experience, reported primary healthcare provider noninvolvement in screening for cognitive impairment. The few that screened revealed that all older patients with cognitive problems that they saw were almost always referred to tertiary health facilities for specialized management. They emphasized that since they did not see such problems frequently, the management of cognitive impairment was not clear; hence they preferred to refer.

“*…even here we rarely see that problem, but when I see one who is obvious with memory problems, I always refer to people who are better at handling that … I send them usually to more senior people in that (cognitive impairment screening) … I don*’*t tamper, I just refer them” **P 12, 40 years old, Female.***

##### 4.1.1.4. Chronic understaffing of the lower facilities

Majority of the participants expressed that chronic understaffing of the primary healthcare facilities resulted into increased workload. This made cognitive impairment screening a lesser priority amidst heavy workload.

“*Of course … according to our health structure, we are supposed to be more … but because we are very few health workers available, I find myself working here at triage, I work at immunization, and in dispensary, at the end of the day, I am very tired … and that (cognitive impairment screening) can't be added on the workload” **P 11, 49 years old, Female.***

#### 4.1.2. Facilitators

##### 4.1.2.1. Role of primary healthcare provider’s involvement

Majority of the participants emphasized the collective responsibility of all health workers (and non-health workers) in identifying all persons with cognitive impairment. This suggested that simple questions and observations at initial assessment could be helpful in identification of cognitive problems.

“*Every health worker is responsible for this screening, because they can use some clues like … sometimes these patients forget the date, the month; so, when you need to ask; “what is the date today?”, “is it morning is it in the afternoon?”, “who is this?” (When s/he comes with a relative), how are you related to this one … then you’ll tell if they have memory problems … ”—**P 2, 32 years old, Female.***

### 4.2. Psychological capability

#### 4.2.1. Barrier

##### 4.2.1.1. Lack of patient awareness about cognitive problems

Primary healthcare providers noted that they only addressed health problems expressed by the patient. As the public considered cognitive problems as part of the normal aging process which didn't require treatment, these health problems would not be addressed. The patient and care taker unawareness of cognitive impairment as a health problem resulted in having the majority of patients not reporting behavioral, social or psychological problems they experienced.

“*…and, in most times, if you ask your patient, “do you have any problem? If the patient responds “I'm okay” at times you don't go to dig deep into the problem, for other complaints.”—**P 5, 38 years old, Male.***

#### 4.2.2. Facilitator

##### 4.2.2.1. Specialized training in cognitive impairment screening

All primary healthcare providers believed that a refresher course or specialized training in cognitive impairment assessment would be important to build their capacity in cognitive impairment screening. Training in using guiding documents and use of screening tools by more experienced persons from higher health facilities would equip them with new skills and knowledge in mental health assessment leading to independence.

“*… like I said workshops, and hands on training … by someone from a big health facility and helps us directly once in a while … so that we get used to it and be independent” **P 3, 32 years old, Female.***

## 5. Domain 2: opportunity

### 5.1. Physical opportunity

#### 5.1.1. Barriers

##### 5.1.1.1. Patient overload

Participants reported high number of patients at the primary healthcare facilities and yet there was a small number of primary healthcare providers. They reported rushing through the assessment process to reduce the queues, and not holistically addressing the patient's complaints.

“*… there are very many patients, and when patients are very many … it is challenging to see one-by-one, you can*’*t be able to assess clearly to identify that problem (cognitive impairment), we end-up seeing each one briefly to address major problems…” **P 3, 35 years old, Female.***

##### 5.1.1.2. Infrastructure shortage

Although, participants preferred special clinics for cognitive impairment screening, it was mentioned that primary healthcare facilities do not have enough physical space to accommodate new clinical assessments, since existing infrastructure is still inadequate for the routine services provided.

“*…There are no special clinics for them; we even don't have adequate space for the usual services we offer daily … So, we don't have a specific clinic for new practices like cognitive impairment screening…” **P 10, 40 years old, Male.***

##### 5.1.1.3. Time constraints

All participants reported the insufficient time to spare per patient because the clinics have handled different co-morbidities. They narrated that it took them more time to assess a patient with more than one disease than those with single disease only. This forced them to refocus their clinical goals from assessing the patient holistically to addressing major illnesses only, hence missing an opportunity for cognitive impairment screening.

“*…we don't have enough time for them. Because we have a lot of patients, and we are few clinical officers and nurses … and sometimes patients may have diseases like Diabetes mellitus, hypertension, some have HIV too. So, you have to look at all their drugs and adjust them accordingly … and counsel them actually, which takes a lot of time and find some are not paid good attention (who may have cognitive issues), because at the end of the day we have to finish the queues…” **P1, 43 years old, Female.***

#### 5.1.2. Facilitators

##### 5.1.2.1. Specialized training in cognitive impairment screening

All primary healthcare providers emphasized the need for a refresher course or specialized training and workshops on cognitive impairment assessment, from more experienced persons. They suggested trainers could be from specialized mental health facilities. They considered acquisition of such skills and knowledge, a move to independent cognitive impairment screening.

“*… if we get workshops, hands-on training … by someone from a higher mental health facility to train us to acquire those skills of cognitive impairment screening and also help us directly once in a while … so that we get used to it and to be independent to do it ourselves” **P 3, 32 years old, Female.***

### 5.2. Social opportunity

#### 5.2.1. Barriers

##### 5.2.1.1. Cognitive impairment screening not prioritized

The majority of participants reported that cognitive impairment screening was not a priority among older persons living with DM and HTN, since primary healthcare providers only put emphasis on control of the medical conditions and managing acute medical complications.

“*For us here, we don*’*t do that very often, it is very hard for you to say “I take priority for cognitive impairment” when the major thing is diabetes or hypertension” **P 8, 47 years old, Male.***

## 6. Domain 3: motivation

### 6.1. Reflective motivation

#### 6.1.1. Barriers

##### 6.1.1.1. Lack of self-confidence about cognitive impairment screening

In this study, majority of primary healthcare providers expressed low confidence in implementing cognitive impairment screening, since they had not received any special training in cognitive screening and in dealing with people with memory problems.

“*You cannot be confident … I just know memory problems; by the way I don't even know what to do with memory problems” **P 12, 40 years old, Female***”.

#### 6.1.2. Facilitators

##### 6.1.2.1. Mentorship from specialized health professionals

Most participants suggested that mentorship in cognitive impairment screening by experienced personnel would facilitate acquisition of special skills. They emphasized that through such mentor-mentee relationships, primary healthcare providers could possibly acquire skills and experience in using screening tools to provide the cognitive impairment screening.

“*In case we are mentored by senior people from higher facilities, they can share experiences, up-dates, and even how to use updated tools….” **P 14, 32 years old, Female.***

“*We need more of mentorship from our senior people from higher facilities, because when they share their experience, it helps us learn to use appropriate materials … this actually makes us suspect such cases easily, because now we know and we can ask our mentors about such problems we see…” **P 14, 32 years old, Female.***

### 6.2. Automatic motivation

#### 6.2.1. Barriers

##### 6.2.1.1. Lack of specific screening guidelines and policy for cognitive impairment screening

Participants revealed that lack of cognitive impairment screening guidelines and screening policy at primary healthcare facilities was a major barrier to screening for cognitive impairment. They added that existing guidelines did not clearly mention how cognitive impairment screening was to be assessed.

“*I've not seen any guidelines showing us what to do about cognitive or memory problems among our old people, maybe I've not read enough, but for the years I've worked, I have not come across it…” **P12, 40 years old, Female.***

#### 6.2.2. Facilitators

##### 6.2.2.1. Motivation to primary healthcare providers

Cognizant of many barriers to cognitive impairment screening like chronic understaffing (capability), patient overload and time constraints (opportunities) and lack of confidence (motivation), majority of the participants emphasized that primary healthcare provider special recognition whether monetary or non-monetary would propel them to willingly learn and adopt new practice of cognitive impairment screening despite the existing challenges. They reported that such actions would be reflective of appreciation to the staff members and would propel them to work.

“*… we also need motivation … maybe in terms of finance or other ways of appreciation for the heavy work we do … although we are paid by the government, but some motivation sometimes is needed to feel that they are liking what you are doing, and we feel appreciated … then you can do something new” **P5, 48 years old, Female.***

## 7. Discussion

In this study, we sought to explore the barriers and facilitators to screening for cognitive impairment among older adults with diabetes mellitus and hypertension by primary healthcare providers in rural Uganda using the Capability, Opportunity, Motivation theory to behavior change. The COM-B frame work has been widely used by various researchers in providing insight into new behavior adoption.

Our study findings highlight knowledge deficits about cognitive impairment screening among primary healthcare providers handling patient populations that are at high-risk for dementia, such as those living with DM and HTN. These results align with a similar study in the same setting that reported knowledge gap regarding the use of the Uganda Clinical Guidelines for provision of mental health services in primary health facilities (19). Similar studies in primary healthcare settings show that primary healthcare providers express low confidence, insufficient competence, and difficulty in recognizing symptoms of cognitive impairment, and that they overlook their importance in health care (20). This reveals that a significant number of diabetic and hypertensive older persons with cognitive impairment in southwestern Uganda are likely to go unnoticed. Due to this, these patients are more likely to progress and present later with severe forms of cognitive impairment. There is need to support primary healthcare providers in diabetic and hypertensive clinics with mental health training in order to equip them with knowledge and skills to screen and identify dementia in its early stages and initiate the appropriate management strategies and timely referrals to halt and retard progression (21).

An important barrier identified in this study was that majority of older adults came alone to seek health care, and as such would not be able to provide adequate information about their cognitive functioning. Other scholars have shown that informal caretakers plays an important role in the comprehensive management of cognitive impairment, and that low participation rates by caretakers limit the overall goal of dementia care (22). In this study therefore, when caretakers do not accompany the older patients to the health facilities it becomes a form of low participation rate of caregivers. This presents an important barrier to practice, since comprehensive screening can only be obtained with proper history from a caretaker other than from the older adult who may not be aware of their declining cognitive state. It is also possible that the lack of caretakers accompanying the older adults to the health facilities, is an example of caregiver burden which could have contributed to the lack of prioritization of screening for cognitive impairment by the practitioners (23). Therefore, community awareness is a tool required to activate community participation to demystify dementia and improve its early detection and planning.

The main facilitator reported by primary healthcare providers was provision of specialized training in the use of screening tools, use of guiding protocols, and mentorship. Similarly, in a review by Aminzadeh *et al*, provision of formal training about dementia case recognition and the use of dementia screening tools was reported to enhance adoption of dementia screening among primary healthcare providers (20). This suggests that health workers in primary care settings require continuous training on cognitive impairment screening through continuous professional developments, and continuous medical education (19). Furthermore, brief assessment tools need to be locally developed, tested and standardized to be integrated into daily clinical practice in order to simplify the screening process (24). Primary healthcare providers should be encouraged to adhere to the Uganda Clinical Guidelines handbook as a guide available for quick identification of dementia or cognitive impairment symptoms (19).

This study addresses a growing need for cognitive impairment screening in low- and middle-income countries where more people live longer with or without comorbidities (e.g., DM, HTN). The results of this study highlight the effect of combination of lack of knowledge about cognitive impairment screening, lack of screening tools coupled with the complexity of cognitive impairment screening compaired to routine diagnostic services like blood pressure and blood glucose measuring. The result of a combination of above factors leads to neglect of cognitive impairment screening in context of high caseloads, resulting into late diagnosis, increased complications, worsening distress to care givers, and poor management of dementia which compromises the quality of life of affected persons. Therefore, exploring barriers and facilitators to cognitive impairment screening is a great step to implementation of sustainable programmes that will support primary healthcare providers to adopt routine cognitive impairment screening in primary health care.

Our sample size was limited to primary healthcare providers; we did not collect opinions of healthcare workers from high level facilities who may have had alternative perspectives on cognitive impairment screening among older adults. Despite this limitation, our strength lies in collecting information from primary healthcare providers with a diverse back ground in terms of experience and qualification using a COM-B frame work which is widely used to understand behavioral change.

## 8. Conclusion

Screening for cognitive impairment by primary healthcare providers at primary healthcare facilities is still poor. Successful implementation of cognitive impairment screening has many provider level barriers and facilitators that require contextual exploration and consideration during service delivery. Therefore, relevant stakeholder involvement is key in adopting suggested facilitators as well as addressing identified barriers. Provision of appropriate training in dementia screening and case definition and detection is crucial in early dementia care enrollment. Mentorship and training in the current practice in the use of specific tools for screening for cognitive impairment is essential in early detection, management and prevention of severe forms of dementia and preventable complications.

### 8.2. Implication to policy and practice

The study has built on previous literature by highlighting the complex determinants of cognitive impairment screening. Across all three levels, multiple barriers and facilitators were identified relating to capacity, opportunity and motivation. To increase cognitive impairment to screening among older adults with diabetes mellitus and hypertension, we should focus on specialized training about cognitive impairment screening, and availing screening tools and guidelines. These seem to be influencing facilitators with everyday practice and could be reinforced by national guidelines, reward and incentive programs based on outcomes. Finally, regarding mode of screening, specific modifications like developing and testing easy to use screening tools specifically targeting cognitive impairment screening among older adults have the ability to reduce multiple barriers such as time constraints, work load, and knowledge deficit. To increase cognitive impairment screening evidence-based approach is needed to design best screening practices.

## Data Availability

The original contributions presented in the study are included in the article/Supplementary Material, further inquiries can be directed to the corresponding author.
