# Supplementary material for: Barriers and facilitators to cognitive impairment screening among older adults with diabetes mellitus and hypertension by primary healthcare providers in rural Uganda
Source: Front Health Serv. 2023 May 30;3:1172943. doi: 10.3389/frhs.2023.1172943 (PMC10266276; doi:10.3389/frhs.2023.1172943)
Supplement: Supplementary file 1 [file Table1.docx]

**Additional file 1: Overview of the Interview guide**

| **Domain** | **Interview question** |
| --- | --- |
| Capability | What do you know about cognitive impairment screening? (Probes)  Do you think it’s important to identify cognitive impairment among older people in primary care? If yes, how do you do it in this facility?  Who can identify persons at-risk of Cognitive impairment? Who is responsible to identify persons with cognitive impairment?  What do you think the guidelines say about screening for cognitive problems?  How often do you think primary care providers should identify cognitive impairment in their elderly patients? (Probes)  What is your practice for identifying cognitive impairment in your elderly patients?  If a patient doesn’t ask you about cognitive problems, how often do you bring them up?  How effective do you feel you are at addressing cognitive impairment with older patients?  What type of support would you need to effectively address cognitive impairment in your older patients? |
| Opportunity | What factors facilitate the implementation of cognitive impairment screening among older patients?  To what extent do your surroundings and working environment facilitate or hinder screening for cognitive impairment among your patients?  (Physical space, workforce, guidance)  What social factors facilitate or hinder you from screening for mental health problems? (supervision, training) |
| Motivation | How much of a priority is screening for cognitive impairment for you among your older patients with DM and/or HTN? What circumstances make it a higher/lower priority?  Do you think you need to screen for cognitive impairments among older persons with diabetes mellitus and/or hypertension?  How easy or difficult do you think is screening for cognitive impairment among older persons?  Do you have any thoughts on how these barriers might be overcome? What type of information or support would you want to have at your fingertips?  Do you think that more training or education on cognitive impairment would be helpful for you? What would be the best way of providing this?  How confident are you in discussing cognitive impairment with older patients with DM/HTN? What would make you feel more confident?  How prepared do you feel to discuss cognitive impairment with your older patients? Is there anything that would make you feel better prepared?  What preparatory steps are there at a health facility level to help you screen for cognitive impairment? (Probes for, how to use available screening tools, measures of encouraging you to screen for cognitive impairment) |
